# Guest Editorial

**Published:** 2016-06-22

**Authors:** AR Prabhakar

**Affiliations:** Associate Dean, Professor and Head, Bapuji Dental College and Hospital, Davangere, Karnataka, India

**DENTAL HOME IN INDIA, WILL IT REMAIN A POTENTIAL REALITY?**

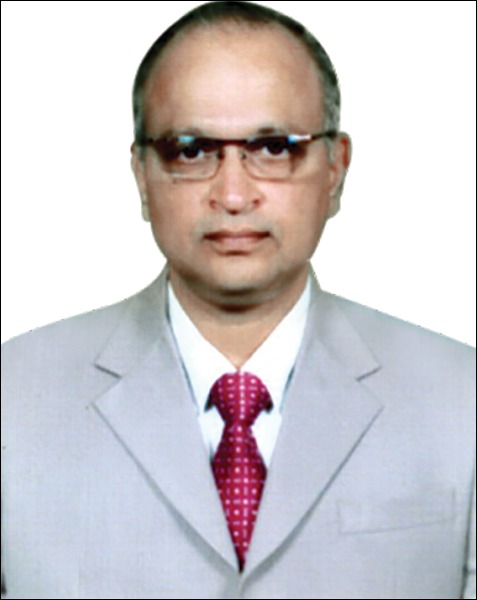

There are new advances happening every single day in the world of dentistry. But is the common man receiving the benefits of our research? In a country like ours, a large segment of the population is rural and is still unaware of the significance of early dental care. Even as oral hygiene awareness increases in the upper segments of the society, a vast part of our social strata has no access to dental care. Early childhood caries (ECC) is the most common chronic dental disease condition in children.

Early childhood caries as we know has far reaching consequences that do not end only at a handful number of decayed teeth or other related issues that we dentists face during oral rehabilitation of these young children. Early childhood caries can lead to reduction in food intake and a subsequent reduction in the ideal body weight of children, loss of school hours and loss of hours in parents’ work. Early childhood caries represents significant financial and societal burden which includes the repeated dental visits for its management and prevention. Does this not call for an immediate need to nip it in the bud? The point I am trying to drive home is that we pediatric dentists must explore and enforce the concept of dental home in India.

Dental home is a concept of coordinated care. Establishment of a dental home begins no later than 12 months of age and includes referral to specialists when appropriate. This means that a significant degree of interaction is required with our pediatrician counterparts and other dental specialties to identify those who need early dental attention and intervention.

In India, one of the obstacles is the perceived lack of qualified pediatric dentists. This can partly be overcome by emphasizing the importance of infant oral health care in the undergraduate syllabus and training the undergraduate dentists in early identification, counseling, preventive and referral protocols.

Establishment of a dental home may not always be feasible at primary health care centers or hospitals. We as a fraternity can employ methods to establish a dental home in the school premises to ensure continued dental care to the children of the school.

There is a profound increase in ECC while on other hand there is also a significant rise in unemployment among dental graduates. The solution lies at the juncture where supply meets the demand. The output of qualified dentists has increased substantially over last decade. India has a dentist to population ratio of 1:10,000 in urban areas and 1:2,50,000 in rural areas. At present less than approximately 5% of dentists are working in the government sector. By inculcating the importance of preventive dental care in our undergraduate students we are not only aiming at accessible oral heath to the economically weaker section but also seeding the thought of prevention of oral disease as a profession in itself. This editorial attempts to open our minds to the surreal future of dentistry that lay ahead. We can play a role in the change only if we wish to. I call out to my fellow colleagues and contemporaries to explore newer ways through which we can bring about a state of better oral and overall health while creating a sense of employment security among the students whom we train to become better dentists for tomorrow.

**AR Prabhakar**

Associate Dean

Professor and Head

Bapuji Dental College and Hospital

Davangere, Karnataka, India

